# A Case Report: Immediate Implant Placement with PRF, Photogrammetry-Guided Workflow, and Monolithic Zirconia Full-Arch Restoration

**DOI:** 10.3390/reports9010008

**Published:** 2025-12-24

**Authors:** Przemysław Bolesław Grzesiak, Adam Aleksander Nowicki

**Affiliations:** 1 Stomatologia Grzesiak Dental Clinic, Żwirki i Wigury 19/1, 43-190 Mikołów, Poland; przemyslav.grzesiak@gmail.com; 2 Diamante Clinica Dental Clinic, Sportowa 48 A/C, 59-300 Lubin, Poland

**Keywords:** immediate implant placement, PRF, photogrammetry, monolithic zirconia, full-arch restoration, case report

## Abstract

**Background and Clinical Significance:** This case presents immediate implant placement combined with PRF (A-PRF+) and a photogrammetry-based workflow, illustrating predictable full-arch implant-supported rehabilitation. **Case Presentation:** Main clinical findings included compromised abutment teeth and patient dissatisfaction with aesthetics. Interventions included extractions, immediate implants, PRF socket management, and digital provisional and definitive restorations. Outcomes demonstrated stable occlusion, satisfactory aesthetics, and high patient satisfaction. **Conclusions:** Immediate placement with PRF and photogrammetry provides reliable outcomes in complex full-arch cases.

## 1. Introduction and Clinical Significance

Full-arch implant-supported rehabilitations are a predictable option for restoring function and aesthetics in patients with partial or complete edentulism. However, managing cases that combine failing tooth- and implant-supported prostheses remains challenging. Compromised abutment teeth, caries, periodontal disease, and unsatisfactory aesthetics often necessitate transition to a fully implant-supported reconstruction.

The decision-making process in such cases is complex, and one must consider the biological, mechanical, and aesthetic factors, as well as the long-term prognosis and patient expectations. Preservation of natural teeth is generally recommended whenever possible; yet, when the prognosis is severely compromised, conversion to a fully implant-supported reconstruction often offers a more predictable long-term solution.

This case report describes the management of a 60-year-old female with long-standing fixed restorations in both jaws. The patient was suffering from failing posterior abutment teeth in the maxilla and dissatisfaction with the aesthetics of her anterior prosthesis. Clinical and radiological evaluation guided the treatment, and it included extractions, immediate implant placement with platelet-rich fibrin (PRF) (A-PRF+) for socket management, and a fully digital prosthetic workflow. The stepwise approach resulted in a stable and aesthetic full-arch reconstruction supported by six maxillary implants.

Photogrammetry-based implant position recording has been introduced to overcome the limitations of conventional intraoral scanning in complete-arch implant cases, where cumulative stitching errors may compromise trueness. Multiple systematic reviews and in vitro studies have demonstrated superior accuracy and reproducibility of photogrammetry-based workflows for complete-arch implant impressions compared with conventional techniques [1,2,3].

Despite its demonstrated accuracy, photogrammetry-based workflows are associated with higher equipment costs, additional clinical steps, and a learning curve that may limit their widespread adoption.

## 2. Case Presentation

A 60-year-old female patient visited us for a routine dental check-up and further treatment planning. The patient had been using well-functioning fixed restorations for many years, including both tooth-supported and implant-supported prostheses in the maxillary and mandibular arches.

The patient was generally healthy, reported no systemic diseases, was not taking any regular medications, and denied smoking.

### 2.1. Mandibular Status

Clinical examination revealed stable and functional restorations in the lower arch. A 4-unit implant-supported bridge was present, supported by two implants in positions 32 and 42. Positions 33–35 and 43–45 were prosthetically reconstructed with tooth-supported blocked crowns fixed on each tooth, respectively. Single implant-supported crowns were present in positions 36 and 46, both in good condition. The patient reported no complaints regarding the mandibular reconstructions.

### 2.2. Maxillary Status

In the upper arch, a 4-unit implant-supported bridge was present in the anterior region, supported by implants in positions 11 and 21, with cantilevers extending to 12 and 22. Segments 23–25 were restored with tooth-supported blocked crowns on teeth 23, 24, and 25. Segments 13–15 were reconstructed with a tooth-supported bridge anchored on teeth 13 and 14, with a distal cantilever in position 15. Additionally, two single implant-supported crowns were located at sites 16 and 26. The clinical evaluation of the peri-implant soft tissues surrounding the previously placed implants revealed favourable conditions, with no signs of inflammation or peri-implant defects. Based on the patient’s records and clinical inspection, the existing implants in the maxilla were identified as ICX implants (diameter 4.1 mm, length 10 mm) (ICX-Implant System; Medentis Medical GmbH, Bad Neuenahr-Ahrweiler, Germany).

### 2.3. The Patient’s Chief Complaint

The patient reported problems associated with the natural abutment teeth supporting the fixed bridges in the posterior maxilla. She complained about halitosis, food retention around these reconstructions, occasionally bleeding gums, and the appearance of black spots on the visible portions of the exposed roots. In addition, she expressed dissatisfaction with the aesthetics of the existing anterior maxillary restoration, particularly regarding the shape and colour of the teeth and wished to improve their appearance.

### 2.4. Clinical Findings

Detailed clinical examination revealed poor marginal adaptation and inadequate crown–tooth marginal adaptation and their abutment teeth at positions 13, 14, 23, 24, and 25. These findings were further complicated by severe root caries and the presence of periodontal pockets, two of which exceeded 5 mm in depth. The overall prognosis for the involved teeth was uncertain and compromised. Such conditions are well documented in the literature as major risk factors leading to the extraction of crowned teeth, with secondary caries, endodontic failures, and periodontal disease being the most frequent reasons for tooth loss under fixed prosthetic restorations [4,5,6].Based on clinical and radiographic findings, the affected teeth structure was insufficient to ensure predictable long-term prosthetic retention, leading to a classification of poor-to-hopeless prognosis according to commonly accepted prosthodontic and periodontal criteria.

The previously placed anterior and posterior implants demonstrated stable peri-implant conditions and favourable prosthetic positioning, allowing their predictable integration into the planned full-arch reconstruction.

The existing occlusal relationships were stable, and the patient reported no temporomandibular joint (TMJ) complaints.

### 2.5. Radiological Examination

**Figure 1:**
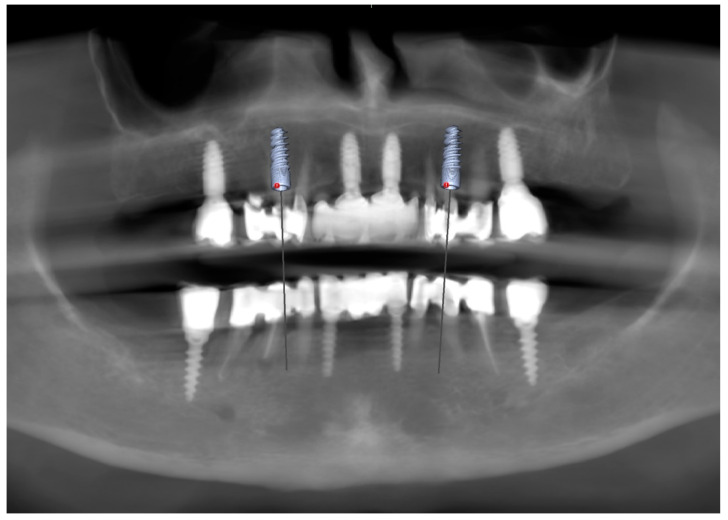
Preoperative panoramic reconstruction from CBCT, showing the initial condition of the existing tooth- and implant-supported restorations in the maxilla and mandible, as well as the visualisation of two planned implants in positions 14 and 24.

To supplement the clinical findings, a cone beam computed tomography (CBCT) scan was obtained (Figure 1) and evaluated. The implants existing in the maxilla demonstrated proper surrounding bone architecture. The compromised status of the remaining teeth was confirmed. In addition, the available bone conditions in the regions of teeth 14 and 24 were assessed to be adequate for future implant placement, particularly in the context of immediate implant insertion. This is in accordance with the literature emphasising that sufficient bone volume, labial plate integrity, and socket morphology, are critical prerequisites for successful immediate implant placement [7,8,9].

### 2.6. Timeline

- Day 0—Initial examination and CBCT.
- Day 1—Treatment planning.
- Day 7—Extractions + immediate implants + PRF + splint and intraoral scanning.
- 24 h—Delivery of 3D-printed provisional full-arch restoration.
- 6 months—Photogrammetry registration and PMMA try-in.
- 6 months—Delivery of the final monolithic zirconia full-arch prosthesis.
- 4 months after definitive prosthesis delivery—Follow-up visit with stable function and aesthetics.

### 2.7. Differential Diagnosis

The differential diagnosis included endodontic failure, secondary caries, and chronic periodontal disease. Correlation of clinical examination with radiographic assessment confirmed the final diagnosis.

### 2.8. Therapeutic Intervention and Outcomes

#### 2.8.1. Treatment Planning and Patient Decision

Several treatment options were presented to the patient. One option involved attempting to preserve and reconstruct the compromised teeth despite their poor mechanical prognosis. However, considering the unfavourable long-term outlook, the relatively high estimated cost, and the patient’s strong desire to improve the aesthetics of the anterior maxilla, she opted for extraction of the compromised maxillary teeth and the fabrication of a new implant-supported full-arch reconstruction.

#### 2.8.2. Treatment Concept

A treatment concept was established to extract hopeless teeth 14, 13, 23, 24, and 25, followed by immediate implant placement in positions 14 and 24. These two additional implants were planned to complete the requirement of a full-arch implant-supported rehabilitation based on six implants, ensuring favourable biomechanics and long-term stability. The treatment plan included the use of the four existing implants. Several recent studies support using six implants in full-arch prostheses, especially when bone anatomy and occlusal loading demand greater support and stability [10,11,12].

In addition to CBCT evaluation, clinical photographs were taken, and intraoral scans of the existing prosthetic work were acquired to preserve the vertical dimension of occlusion, phonetics, and aesthetics as a digital reference (biocopy). A Dexis intraoral scanner (Dental Imaging Technologies Corp., Hatfield, PA, USA) was used. These datasets were planned to be merged later with implant position scans to provide continuity between the preoperative and postoperative stages.

**Figure 2:**
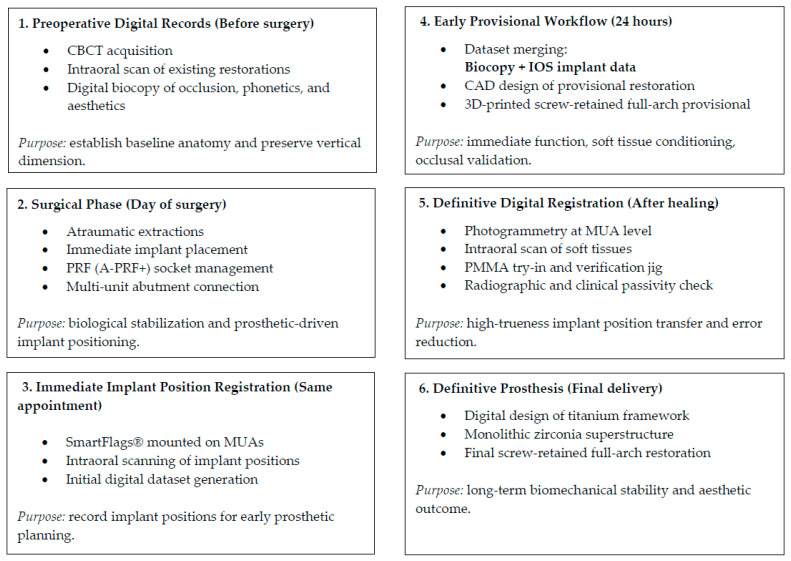
Stepwise digital workflow illustrating the integration of CBCT, intraoral scanning, photogrammetry, and prosthetic design in a full-arch implant rehabilitation. Preoperative biocopy data were merged with implant position records obtained via scan flags and photogrammetry, enabling accurate transfer of occlusal, functional, and soft-tissue information from the provisional to the definitive restoration.

The overall digital workflow applied in this case is summarised in Figure 2.

#### 2.8.3. Surgical Phase

Prior to surgery, a transparent acrylic tooth-supported splint (Ercodur^®^, Erkodent, Pforzheim, Germany) was fabricated, incorporating acrylic teeth 13–15 and 23–25. The purpose of this provisional device was to temporarily replace the teeth to be extracted.

**Figure 3:**
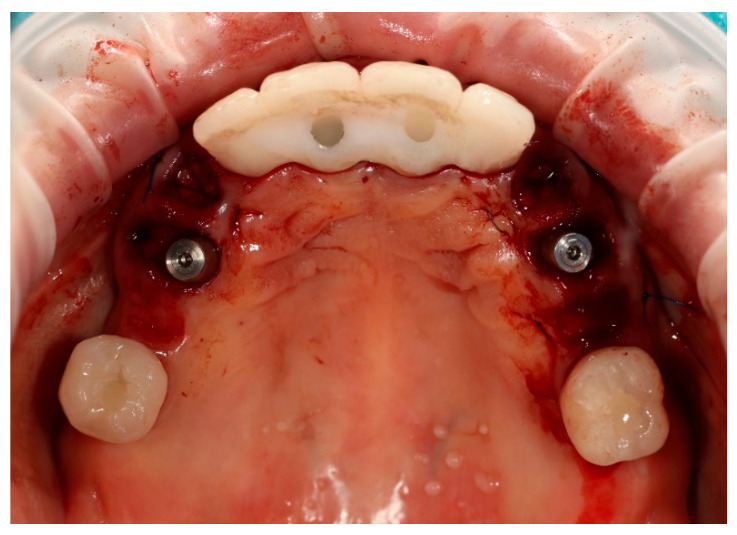
Remaining prosthetic restorations still in place, with visible post-extraction sockets and the two immediately placed implants in positions 14 and 24.

The surgical procedure was performed under local anaesthesia. In the first stage, teeth 13, 14, 23, 24, and 25 were atraumatically extracted. Immediately thereafter, two ICX Active implants (diameter 3.75 mm, length 12.5 mm) (ICX-Implant System; Medentis Medical GmbH, Bad Neuenahr-Ahrweiler, Germany) were inserted into the post-extraction sockets in positions 14 and 24. Excellent primary stability exceeding 35 Ncm was achieved for both implants. Two ICX Active implants were placed following the manufacturer’s drilling protocol, adapted to the clinical conditions of the post-extraction sockets (Figure 3).

The post-extraction sockets were filled with platelet-rich fibrin (PRF) membranes, which were carefully adapted to fill the gaps between the immediately placed implants and the buccal socket walls. Platelet-rich fibrin was prepared using the advanced PRF+ (A-PRF+) protocol, with low-speed centrifugation at approximately 1300 rpm for 8 min, in accordance with standard A-PRF+ preparation guidelines. Low-speed centrifugation protocols forming the biological basis of A-PRF+ have been shown to enhance the biological properties of platelet-rich fibrin, including increased leukocyte content and sustained growth factor release, thereby supporting early soft tissue healing and contributing to bone regeneration [13].

Centrifugation was performed using a dedicated clinical centrifuge according to the A-PRF+ low-speed protocol; rpm values may vary depending on rotor radius.

The use of PRF as a gap-filling material in immediate implant placement has been reported to enhance soft tissue healing and support ridge preservation [14,15]. Clinical trials have further shown that PRF reduces marginal gingival recession and promotes favourable soft tissue contouring around immediately placed implants, particularly in the aesthetic zone [16,17]. Stabilising sutures 5–0 PTFE were placed.

**Figure 4:**
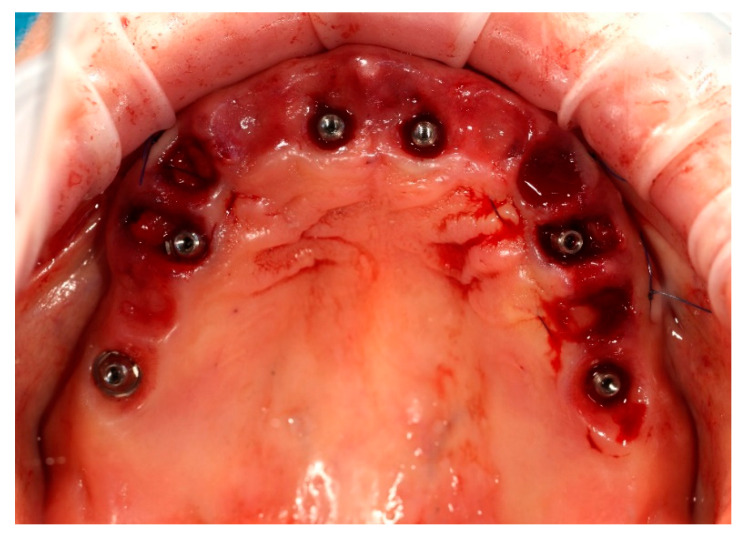
Multi-unit abutments secured onto all six supporting implants, including the newly placed implants at positions 14 and 24, following removal of the existing restorations.

Simultaneously, the existing implant-supported crowns in positions 16 and 26, as well as the anterior implant-supported bridge, were unscrewed from the supporting implants. Multi-unit abutments were then selected and secured onto all six supporting implants, including the newly placed implants at sites 14 and 24 (Figure 4).

**Figure 5:**
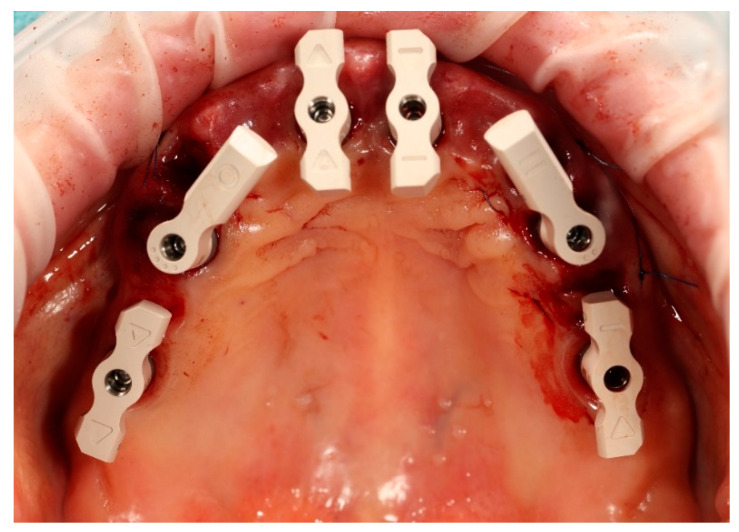
Apollo^®^ scan flags mounted on the multi-unit abutments in the maxilla prior to intraoral digital impression.

Scanbody SmartFlags^®^ (Apollo Implants Components GmbH, Pforzheim, Germany) were mounted to the multi-unit abutments, and an intraoral digital impression of both arches was obtained using an intraoral Dexis scanner (Figure 5). After scanning, the SmartFlags^®^ and multi-unit abutments were removed from four ICX implants, and the original restorations were reassembled. The newly placed ICX active implants were left with multiunit abutments and secured with corresponding healing caps.

**Figure 6:**
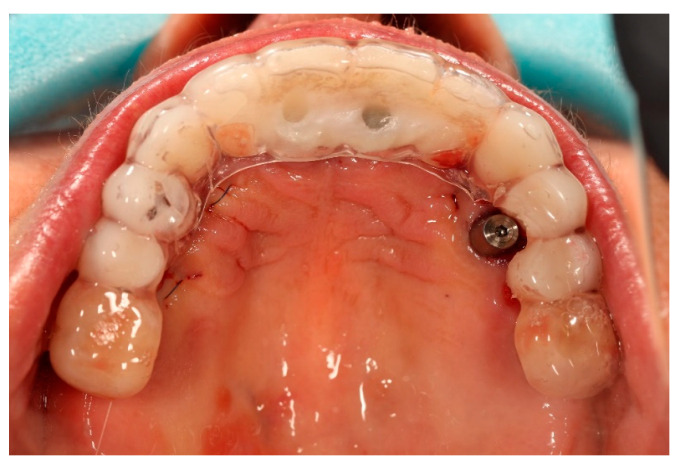
Healing caps inserted onto the newly placed implants MUA at positions 14 and 24, with the original restorations reassembled and the acrylic splint positioned in situ (occlusal view).

**Figure 7:**
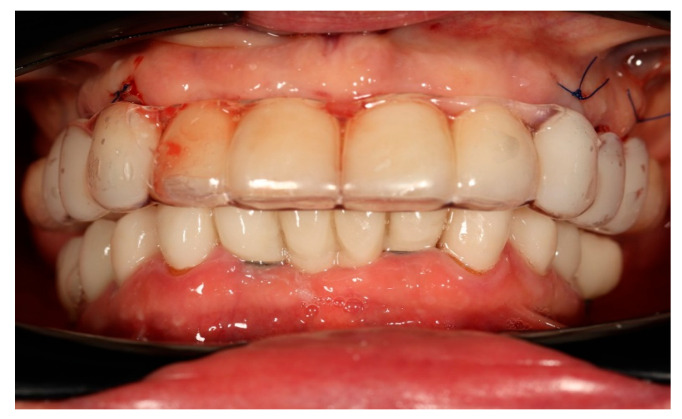
Healing screws inserted into the newly placed implants at positions 14 and 24, with the original restorations reassembled and the acrylic splint positioned in situ (front view).

At the end of the procedure, the transparent acrylic splint was fixed on the remaining prosthetic restorations, ensuring that no contact or pressure was applied to the healing screws at positions 14 and 24 (Figure 6 and Figure 7).

The transparent acrylic splint was fabricated with the Erkopress pressure-forming system (pursuant to the manufacturer’s protocol) prior to surgery. It served exclusively as a short-term aesthetic and protective device during the initial healing period and was not incorporated into the digital workflow.

Postoperatively, the patient was properly instructed and prescribed antibiotics (amoxicillin/clavulanic acid) and anti-inflammatory medication.

### 2.9. Prosthetic Phase

#### 2.9.1. Early Prosthetic Phase

This provisional acrylic splint was worn for approximately 24 h and allowed the patient to maintain aesthetics and function during the first day after the procedure.

The existing occlusal relationships were stable, and the patient reported no temporomandibular joint (TMJ) complaints. Based on the intraoral scan obtained with SmartFlags^®^ and the digital impression of the opposing arch, a virtual model of the maxilla was generated. The dataset was transferred to the dental laboratory for prosthetic planning.

The next appointment was scheduled after 24 h. At the 24 h follow-up, the clinical situation was considered appropriate for proceeding with the provisional restoration. The existing prosthetic restorations were unscrewed from the implants, and the previously selected multi-unit abutments were mounted on all four supporting implants, excluding the newly placed ones, where the closing caps were removed.

**Figure 8:**
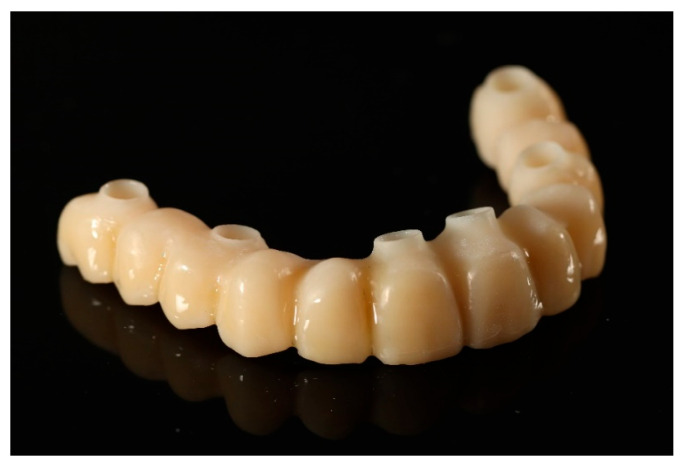
A digitally planned, 3D-printed full-arch provisional restoration ready for assembly.

Based on the merged datasets (biocopy + SmartFlags^®^ scan), the provisional screw-retained, implant-supported full-arch prosthesis was designed in Exocadsoftware (Exocad GmbH, Darmstadt, Germany) and subsequently fabricated using OnX Tough 2 resin (SprintRay Inc., Los Angeles, CA, USA) by means of 3D printing. The intaglio (tissue-facing) surface was polished [18], while the labial surface was characterised and glazed to improve aesthetics (Optiglaze Clear, GC International AG, Luzern, Switzerland). As demonstrated in the accompanying clinical photographs, minor superficial cracks in the glaze layer are visible on the external surface of the prosthesis (Figure 8).

**Figure 9:**
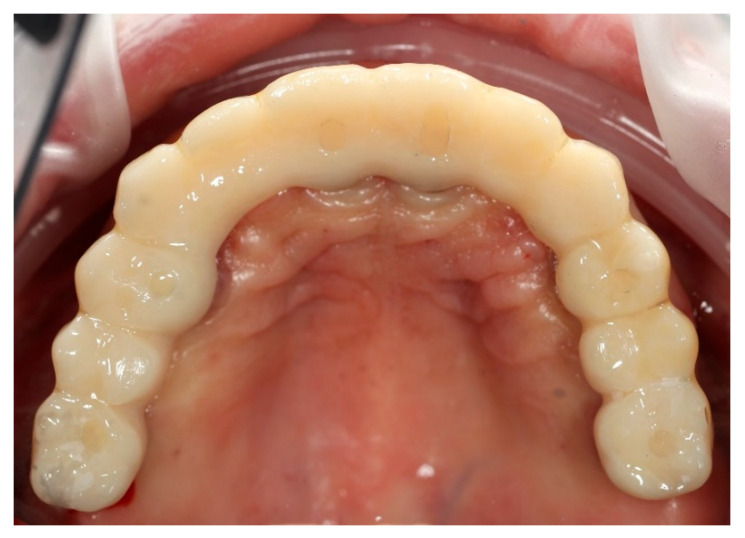
Digitally planned, 3D-printed full-arch provisional restoration delivered early and fixed to the multi-unit abutments with screws, shown in situ (occlusal view).

**Figure 10:**
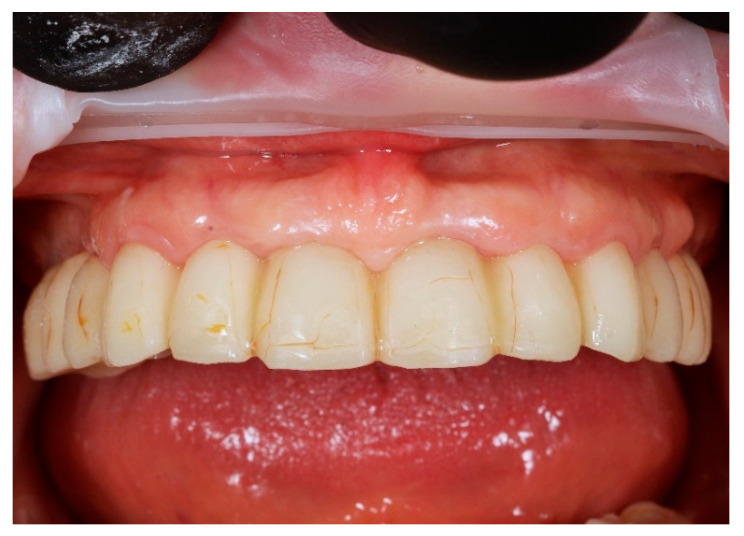
Digitally planned, 3D-printed full-arch provisional restoration after the healing phase and fixed to the multi-unit abutments screws, shown in situ (front view).

Finally, the prosthesis was delivered by fixing to the multi-unit abutments with screws. Clinical adjustments were performed to ensure perfect fit, occlusion, phonetics, and aesthetics. The patient reported immediate improvement in comfort and satisfaction compared to the acrylic splint (Figure 9 and Figure 10).

This early restoration provided stable occlusion, acceptable aesthetics, and proper phonetics while allowing progressive soft tissue conditioning. It also constituted the foundation for definitive prosthetic planning. The patient was instructed on hygiene maintenance and placed on a soft diet during the initial healing period.

**Figure 11:**
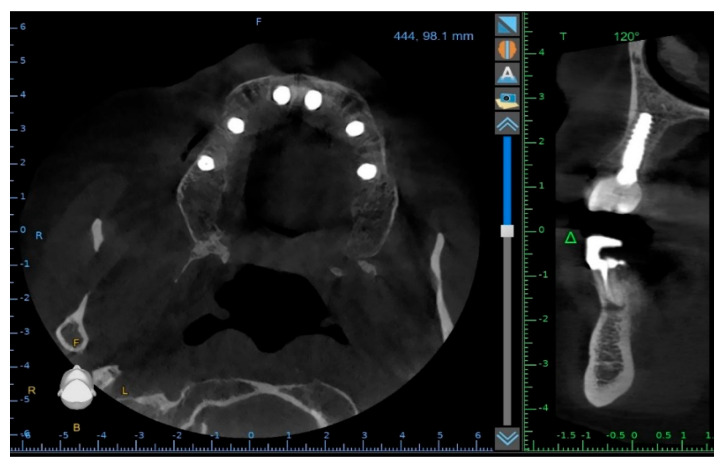
Control CBCT scan obtained after the delivery of the early full-arch provisional restoration.

**Figure 12:**
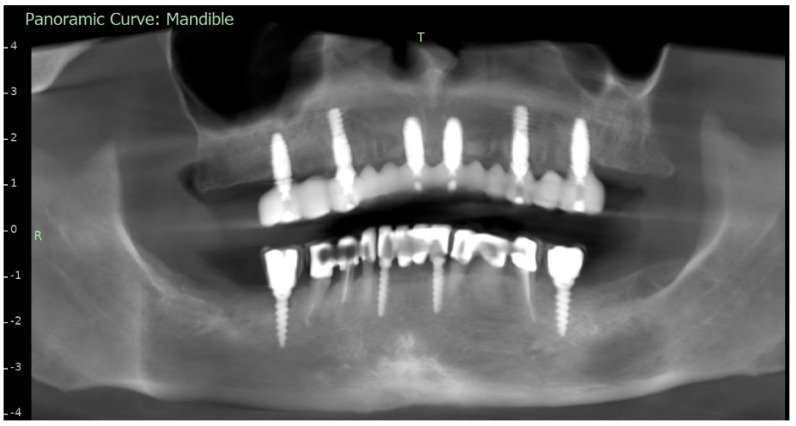
Control CBCT scan obtained after the delivery of the early full-arch provisional restoration.

To verify the implant positions and the accuracy of the prosthetic fit, a control CBCT scan was obtained after the delivery of the early provisional restoration. Radiological evaluation confirmed stable positioning of the implants and correct seating of the prosthesis (Figure 11 and Figure 12).

#### 2.9.2. Final Prosthetic Phase

After an uneventful six-month healing period, the patient made no complaints, presented stable occlusion and satisfactory phonetic and masticatory function. Clinical examination confirmed centric relation, as the verification of the maxillo-mandibular relationship. Centric relation is acknowledged as a critical step in the establishment of new occlusion within implant-supported complete-mouth rehabilitations [19].

**Figure 13:**
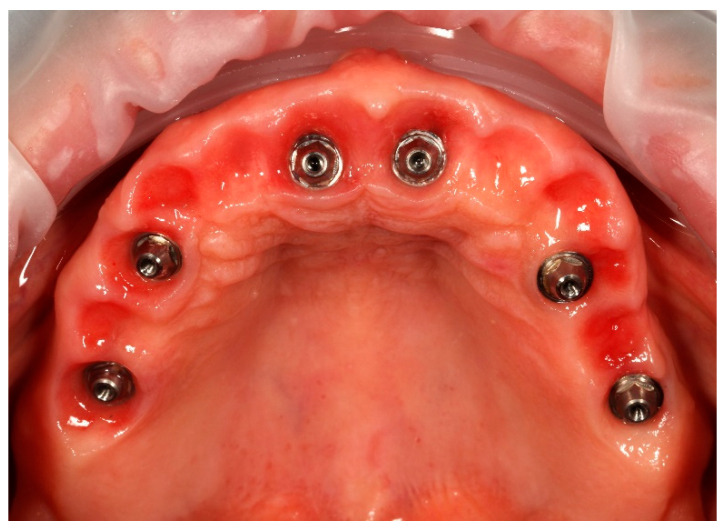
Multi-unit abutments in situ after the removal of the provisional bridge, showing well-healed peri-implant soft tissues contoured by the pontics of the temporary restoration.

Upon removal of the provisional bridge from the multi-unit abutments, well-conditioned soft tissues were observed, adequately shaped by ovate pontics and corresponding to the requirements of the FP1-type full-arch prosthesis (Figure 13). No signs of inflammation or adverse response were noticed. As highlighted in the literature, provisional restorations are considered crucial for conditioning peri-implant soft tissues, as they help to establish the emergence profile and papillae prior to the definitive prosthesis being fitted [20].

Minor aesthetic changes according to the patient’s wishes and clinical assessment were noted. Clinical photographs were taken with the provisional restoration in situ to facilitate minor adjustments in the aesthetics of the definitive prosthesis.

**Figure 14:**
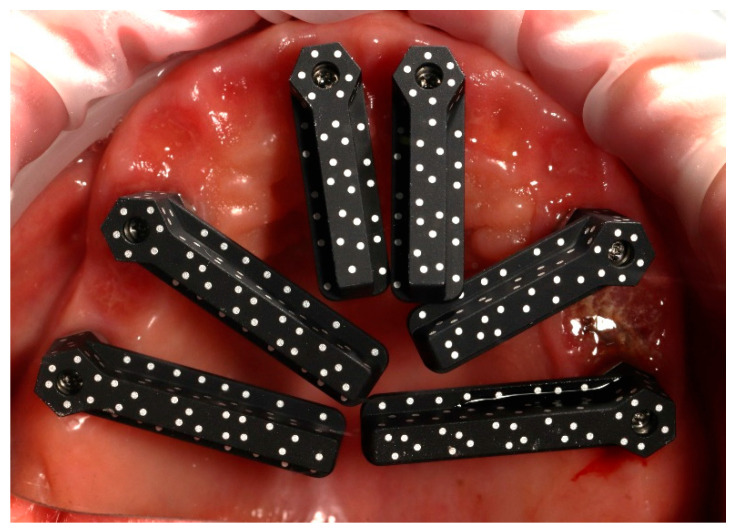
Shining 3D scan flags mounted on the multi-unit abutments in the maxilla prior to the intraoral scanning procedure.

An additional intraoral registration was performed at the level of the multi-unit abutments (MUAs) using scan flags (Shining 3D Tech Co., Ltd., Hangzhou, China) in combination with photogrammetry (Figure 14). This workflow allows the three-dimensional implant positions to be recorded with high trueness and precision, thereby reducing the risk of cumulative errors inherent to conventional intraoral scanner (IOS) stitching, as highlighted in recent in vivo and in vitro studies [2,21].

The Shining3D scan flags are designed to capture IOS and photogrammetry data simultaneously within a controlled and stable scanning pathway. Their integrated fiducial markers enable the software to perform a dual alignment process, cross-referencing implant positions between photogrammetry data, IOS meshes, and the scanned soft tissues. The resulting dataset provides a unified and highly accurate digital model of the clinical situation, which can efficiently be exported to the dental laboratory for prosthetic planning and design [3].

In parallel, the provisional restoration was scanned, and the datasets were merged with CBCT in Exocad software. This workflow allowed for the faithful transfer of the verified occlusal relationships, phonetics, and soft tissue contours which were accepted by the patient during the provisional phase and thus served as a reliable reference into the definitive design.

**Figure 15:**
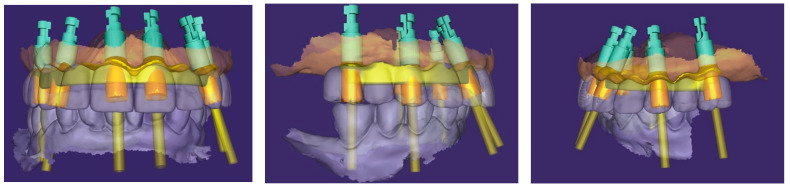
Digital project of the maxillary arch in Exocad. The images show the planned implant positions with their axes, corresponding multi-unit abutments, and the designed metal framework (yellow overlay) for full-arch prosthetic restoration (front, right and left side view).

Based on this integration, a screw-retained titanium bar was digitally designed (Figure 15) and subsequently milled. The passivity of the framework was clinically verified with a PMMA try-in prosthesis and radiographic evaluation.

#### 2.9.3. Passivity Check

To confirm the passive fit of the definitive titanium framework, a resin verification jig was fabricated and sectioned intraorally. The sections demonstrated complete passivity when reseated and rejoined, confirming the accuracy of the master model, consistent with recent in vitro findings on the accuracy of verification jigs [22]. Subsequently, the titanium framework was tested intraorally and showed a stable and passive adaptation to the implants.

A panoramic radiograph was obtained with the framework in place, which confirmed accurate seating of the abutments and framework without visible misfit. Radiographic assessment has been reported as a useful adjunct to clinical methods, although not a substitute for mechanical testing [23]. Additionally, the Sheffield test (one-screw test) was performed by tightening a single distal screw and verifying complete passive seating of the framework at the remaining implant sites. This method has been recognised as a reliable and straightforward clinical approach for detecting misfit [24]. Both assessments confirmed the passivity of the titanium framework.

**Figure 16:**
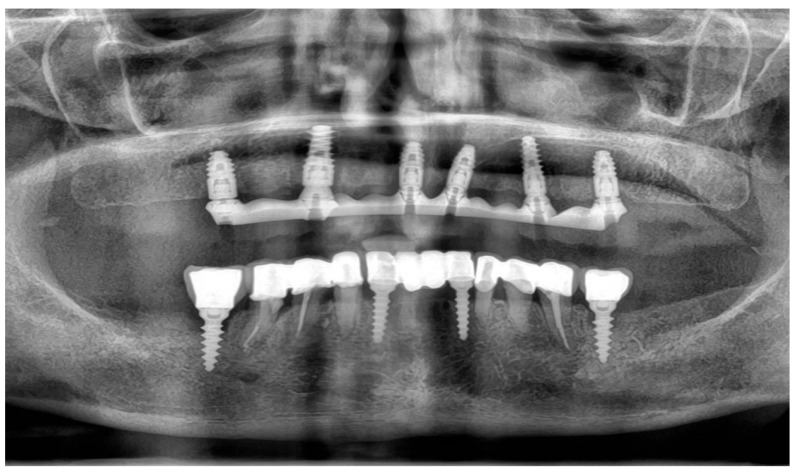
Clinical try-in of the PMMA provisional restoration on the definitive titanium framework to verify the fit, occlusion, and aesthetics.

Next, a clinical try-in of the PMMA provisional restoration on the definitive titanium framework (Figure 16) was carried out. This step allowed verifying the prosthetic fit, occlusion, phonetics, and aesthetics. The clinical role of provisional PMMA restorations in evaluating occlusion and aesthetics prior to the fabrication of the definitive restoration has been emphasised in the literature [25,26]. The restoration was confirmed to be clinically acceptable and was approved by the patient.

#### 2.9.4. Definitive Restoration

**Figure 17:**
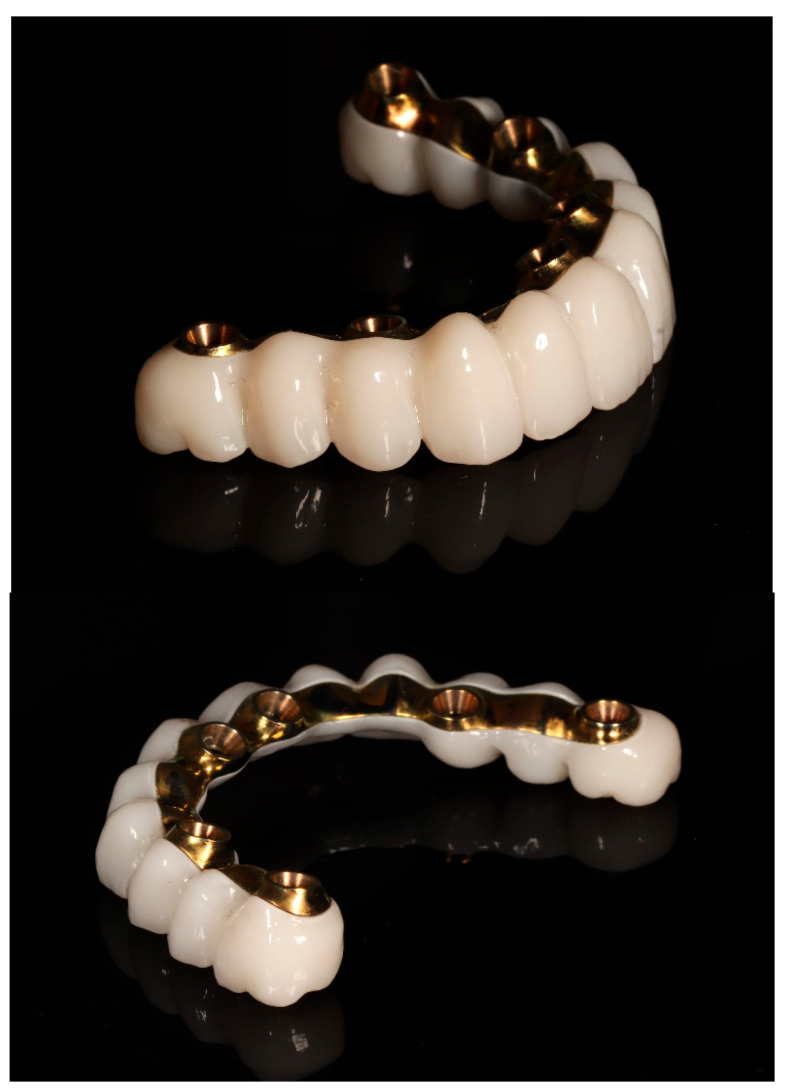
Definitive full-arch prosthesis fabricated on the titanium framework (material: monolithic zirconia).

Following the confirmation of the passive fit, and a successful clinical try-in, the definitive full-arch prosthesis was fabricated as a monolithic zirconia superstructure on the same titanium framework (Figure 17) and delivered to the patient.

A custom-milled titanium framework provided rigidity and long-term stability. A monolithic full-contour zirconia superstructure was manufactured and bonded onto this framework, ensuring both mechanical strength and enhanced aesthetics. Recent reports confirm that zirconia-on-titanium combinations offer predictable outcomes in terms of aesthetics, function, and biological response [27].

The definitive prosthesis was delivered to the patient after laboratory verification. Intraoral evaluation confirmed accurate seating, passive fit, and proper occlusal relationships. Phonetics and aesthetics were reassessed and found to be satisfactory, consistent with previous retrospective data showing high survival rates and patient satisfaction with zirconia-based full-arch prostheses [28]. A panoramic radiograph was obtained at the time of prosthesis delivery to confirm the correct seating of the framework–zirconia assembly on all supporting implants, as well as to verify stable peri-implant bone conditions.

**Figure 18:**
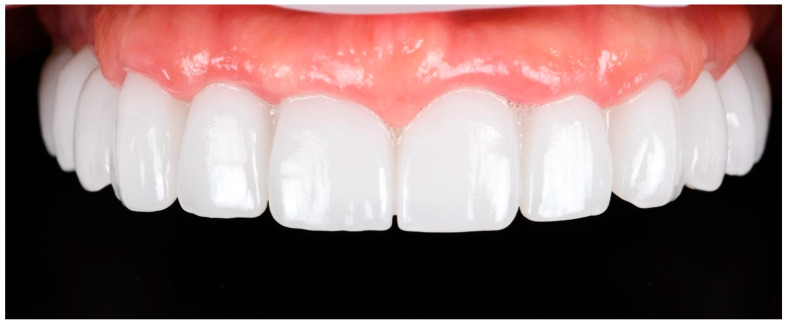
Extraoral frontal view of the definitive full-arch prosthesis in situ, demonstrating the final aesthetic outcome.

**Figure 19:**
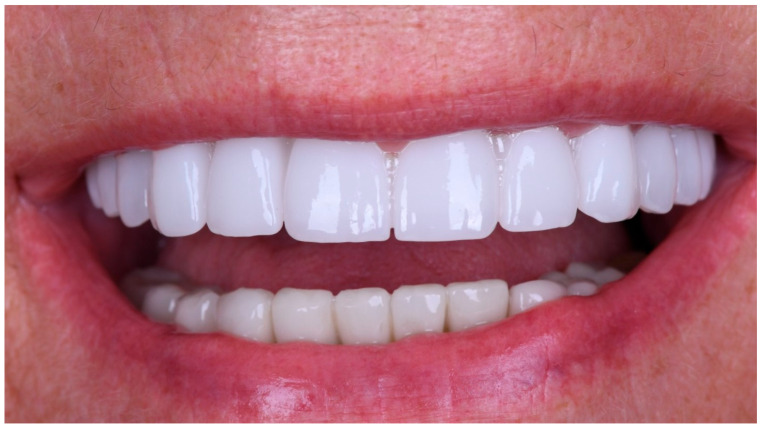
Extraoral smile view of the patient with the definitive restoration in situ.

The restoration was torqued in line with the manufacturer’s specifications, and the screw access channels were sealed with composite resin. The definitive clinical situation in the patient’s mouth is shown in Figure 18 and Figure 19.

Static and dynamic occlusion were subsequently assessed using a digital OccluSense pressure analysis system (Bausch, Hainspitz, Germany).A follow-up was scheduled after 4 months, and the prosthesis demonstrated stable function and satisfactory aesthetics. The patient was fully satisfied.

At the 4-month follow-up, peri-implant tissues were clinically healthy, with no signs of inflammation or suppuration. Clinical and routine radiographic assessment performed during follow-up revealed no pathological findings. The patient reported high satisfaction with function and aesthetics. No biological, mechanical, or technical complications were observed during the follow-up period. The patient was also seen at a later recall visit, during which stable function and satisfactory aesthetics were maintained.

## 3. Discussion

The decision to retain the existing anterior and posterior implants was based on their favourable positioning, stable peri-implant conditions, and adequate prosthetic distribution, which allowed their predictable integration into the new full-arch design. The use of six implants provided improved load distribution and biomechanical stability, particularly considering the patient’s occlusal scheme.

The main clinical challenges included managing multiple datasets across different time points and ensuring accurate alignment between IOS, photogrammetry, and CBCT data. These challenges were addressed through the use of standardised scan flags, consistent reference geometries, and verification steps including PMMA try-in and radiographic controls.

Strengths and Limitations: The main strength of this case is the integration of immediate implant placement, PRF, and a photogrammetry-based digital workflow. The main limitations of this case include the relatively short follow-up period and the inherent limitations of a single-case design, which restrict generalizability. Additionally, the digital workflow applied requires operator experience and access to advanced digital equipment, which may not be universally available.

Take-Home Message: Immediate implant placement with PRF and photogrammetry provides stable, predictable full-arch outcomes even in patients transitioning from failing tooth- and implant-supported prostheses.

## 4. Conclusions

From the patient’s perspective, the treatment resulted in improved comfort and confidence, with the final prosthesis perceived as natural and well tolerated.

## Data Availability

The original data presented in this study are available on reasonable request from the corresponding author. The data are not publicly available due to privacy concerns.
